# Ready to collaborate?: medical learner experiences in interprofessional collaborative practice settings

**DOI:** 10.1186/s12909-020-1992-1

**Published:** 2020-03-23

**Authors:** Ann Ding, Temple A. Ratcliffe, Alanna Diamond, Erika O. Bowen, Lauren S. Penney, Meghan A. Crabtree, Kanapa Kornsawad, Christopher J. Moreland, Sean E. Garcia, Luci K. Leykum

**Affiliations:** 1 University of Texas Health San Antonio, San Antonio, TX USA; 2 Brown University, Providence, RI USA; 3 University Health System, San Antonio, TX USA; 4 South Texas Veterans Affairs Health System, San Antonio, TX USA; 5 Colorado State University, Fort Collins, CO USA

**Keywords:** Interprofessional collaboration, Interprofessional education, Graduate medical education, Undergraduate medical education

## Abstract

**Background:**

Interprofessional collaborative practice (IPCP) offers great potential to improve healthcare. Increases in IPCP will require educating learners in authentic IPCP settings and will generate opportunities and challenges.

**Methods:**

In January 2015, we implemented an IPCP model called Collaborative Care (CC) for hospitalized adult medical patients. We explored learner perspectives regarding their educational experiences. We deductively coded transcripts from semi-structured interviews with medical learners. Data related to educational experiences were thematically analyzed.

**Results:**

Twenty-four of 28 (85.7%) medical learners rotating on CC from January to May 2015 completed interviews. Subsequent inductive analysis of these interviews identified four themes: *Loss of Educational Opportunities during Rounds*, *Feelings of Uncertainty during New Situations*, *Strategies for Adaptation*, and *Improved Communication with Patients and the Team.*

**Conclusions:**

Increased implementation of IPCP will lead to a greater number of learners being exposed to authentic IPCP settings and will generate opportunities and challenges. Though learners perceived improved communication skills in an IPCP model, they also described loss of profession-specific learning opportunities and feelings of uncertainty. These findings corroborate the need for novel teaching methods aligned with IPCP clinical learning environments and educational assessment strategies that reflect attainment of both profession-specific and interprofessional competencies.

## Background

Interprofessional collaborative practice (IPCP) settings are clinical environments in which providers from more than one profession work together in a coordinated manner, developing care plans with each other and with patients and families [1]. These collaborations are increasingly viewed by policy stakeholders, professionals, patients and families as key to improving healthcare [2–5]. Numerous IPCP models have been established across healthcare; however, IPCP implementation can vary widely in terms of overall structure and degree of interprofessional integration, leveling of hierarchy, and patient and family integration [6–8]. Additionally, implementation can be challenging [9–12].

Concurrent with increased emphasis on the importance of IPCP has been increased focus on interprofessional education (IPE). The growth in IPE activity to date has largely occurred in preclinical contexts. Ultimately moving IPE from the preclinical classroom to authentic IPCP environments is recognized as necessary to achieve widespread adoption of IPCP [3, 4, 13, 14]. Environments where IPCP and IPE coexist have been termed “nexuses,” and national calls for establishment of these educational clinical environments theorize benefits for learners [4].

Although there is a growing literature supporting preclinical and outpatient-based clinical IPE initiatives, IPE initiatives in inpatient IPCP settings are described less in the literature [15]. This gap includes few studies focused exclusively on medical learners in inpatient IPCP settings [16]. Thus, we have limited knowledge of medical learners’ (i.e., internal medicine residents and medical students) perceptions of inpatient IPCP clincal learning environments, particularly ones that intentionally level hierarchy and are thoroughly patient-partnered.

Compared with traditional educational settings (e.g., in a classroom), medical student clinical rotations (e.g., medicine clerkships), and especially residency training, are aligned with workplace learning theories of individual and social cognition. Workplace learning illuminates the largely uncodified dissemination of tacit and formal knowledge that occurs through ritualized teaching and learning in workplace settings [17]. Potential inherent tensions exist between traditional models, where medical learners are directly mentored by more senior, physicians, and IPCP environments, where medical learners are expected to integrate with and learn from a variety of professions. How will medical learners respond to an integrated model that also prioritizes the needs of patients and other professions? Will medical learners be able to develop fully as medical professionals within interprofessional environments?

To begin to answer these questions, as part of quality improvement with an IPCP implementation, we explored medical learner perspectives of educational experiences in an authentic, immersive IPCP environment (see Table 1 and description below). Our goal is to use these insights to promote more effective inclusion and immersion of medical learners in inpatient IPCP settings.

**Table 1:** “Usual” Hospital Care vs. Collaborative Care

“Usual” Hospital Care	Collaborative Care
Separate professional workflows	Each profession’s schedule is aligned to promote interprofessional collaboration throughout the day
Physician-led / centered rounds	Patient / family-partnered rounds with interprofessional team^a^
Daily care plan not necessarily transparent / accessible	Daily care plan developed /documented in patients’ rooms on a white board
No dedicated time to reflect on performance	Daily team reflection sessions
Interprofessional component “added on” to workflow	Interprofessionalism supports workflow

^a^ Dialogue at the bedside follows a semi-scripted, yet dynamic process. Each discussion starts with introductions, followed by a review of overnight events and then a problem-based discussion; finally, a safety checklist is reviewed, and the care plan is reinforced at the conclusion. Throughout each discussion, all team members, including patients and families, are encouraged to contribute, and plans emerge that are truly patient-partnered. Care plans are documented on a structured dry erase board in the patient’s room

## Methods

We analyzed data from semi-structured interviews of medical students and internal medicine residents rotating on an IPCP team over the first 5 months of our IPCP implementation, looking for themes related to the impact of the rotation on their perceptions of their educational experiences.

### Study setting

In January 2015, we began implementing an IPCP model called Collaborative Care (CC) for hospitalized adult medical patients on our General Medicine teaching services in a partnership between University of Texas Health San Antonio (UTHSA) and University Hospital (UH) [18, 19]. UH is a Level 1 trauma center with 670 acute-care beds and is one of two main teaching hospital partners for UTHSA. The typical census of the teaching team is 15–17 patients per day.

### IPCP implementation

In CC, the interprofessional team typically consists of one attending internal medicine physician, nurses, one physical therapist, one pharmacist, one social worker, two medical students in year 3 or 4 of a 4 year undergraduate medical degree, two medical interns, and one medical resident in the 2nd or 3rd post-graduate year. In this model, each profession’s workflows are adjusted to allow the team to spend more time together; collaborative, interprofessional bedside rounds replace traditional physician rounds; and the team meets at prespecified times to reflect on team performance and review clinical updates. Table 1 compares CC with more traditional inpatient care models.

### Semi-structured interviews

As part of our implementation of CC, we assessed patient, provider, and learner experiences in an effort to inform and improve our implementation. Semi-structured interview questions were developed in the fall of 2014 by our CC steering committee, which included representation of all provider groups as well as patients and families. Questions were open-ended and involved asking medical learners what they liked or did not like about the experience, how they believed CC differed from traditional practice, how learners’ relationships with patients and other providers were affected, and how the model impacted their learning experience. Suggestions for improvement were also solicited. For this analysis, we used learner responses to questions related to their learning experiences. The full list of questions used is shown in Supplementary file 1.

### Study participants

Medical students, interns, and residents rotated through CC for four-week periods. Interviews were conducted during the last week of each participant’s rotation. From January to May 2015, 28 learners rotated through the CC team, and all were invited to participate in an interview. Interviews were conducted individually, in pairs, or in groups of three interviewees. All interviews were performed by a trained interviewer, and all interviews were audio recorded and transcribed.

### Ethical considerations

The formative evaluation of our Collaborative Care improvement initiative was deemed not to be human subjects research by the UT Health San Antonio Institutional Review Board.

### Analysis

We conducted deductive and inductive qualitative analysis to identify learner experiences and perceptions [20]. We constructed an initial code book with domains drawn from interprofessional learning and collaborative practice (e.g., education, clinical workflow, role of patient and family). Transcripts were assigned to pairs of team members to code within MaxQDA Analytics Pro version 12. To ensure each transcript was reviewed from multiple perspectives and improve cross-team learning, each pair generally consisted of a member with clinical (e.g., physician) and a member with qualitative research (e.g., PhD researcher) experience. Medical student researchers had not yet experienced collaborative care firsthand. Each pair analyzed transcripts independently and then met to resolve discrepancies. In addition, all team members met in a large group setting three times to ensure consistency in initial analysis across transcripts.

AD and TR separately conducted thematic analysis [21] on a subset of learners’ responses coded as “Educational Impact” and “Ideal Vs. Real Experiences.” They met with a subset of the team (LL, EB, LP) to discuss their findings. The subgroup refined the themes in two consensus-building meetings. As part of the participatory/inclusive nature of this quality improvement effort, these themes were also shared with stakeholders (interprofessional CC steering committee) to inform ongoing efforts to improve educational experiences.

## Results

Twenty-four learners (85.7%; 24/28) completed interviews. Trained interviewers conducted the 17 interviews with individuals (*n* = 12), in pairs (*n* = 6), and in groups of three (*n* = 6). Of the 24 learners, 12 were medical students, 7 interns, and 5 residents.

Our inductive analysis identified four themes from 73 learner responses related to their educational experiences: *Loss of Educational Opportunities on Rounds*, *Feelings of Uncertainty during New Situations*, *Strategies for Adaptation*, and *Improved Communication with Patients and the Team*. These themes are summarized below, with specific examples of responses shown in Table 2.

**Table 2:** Themes and Examples of Themes from Interviews with Medical Learners

Theme	Examples
Loss of Educational Opportunities During Rounds	- “You are losing that whole, bouncing off ideas with members of the team, bouncing off ideas with the attending. I think a lot of that is lost, and a lot of valuable learning comes from those experiences.” – Intern 1 - “I feel like there was less teaching going on for the most part.” – Resident 1 - “You do miss out on the formal presentations. The med students do not get the practice, which I think is a pretty big skill. […] Bedside teaching is also gone and lost because you are trying so hard to just simplify patients’ problems into one or two things.” – Intern 1 - “I feel like the teaching has at first really struggled […]. You don’t have that time to sit and discuss things because you are with the whole team. […] I think that is a challenge as a med student and because you know the whole reason why we come is to try to learn how to manage these illnesses and things, you know, not so much how to work with the caseworker. It is good to learn that, [but] right now that is not necessarily what our goal is.” – Student 1
Feelings of Uncertainty during New Situations	- “I cannot say I think it is pancreatic cancer in front of the patient.” – Student 2a - “I do not want to derail the whole process because I have a question about something. […] I think that it kind of hinders my freedom to kind of ask and inquire because I do not want to make it seem like I am questioning somebody’s approach to their care in front of the patient. […] So just to […] not hold [up] the process, I do not ask lots of questions.” – Student 3 - “I think there were times where maybe I would like to ask a question that if it was just med students, interns, residents and attending, maybe I would have brought up that question and we could have discussed a little bit further. But actively in front of the patient, with other team members there, I probably did not bring it up until we walked out of the room.” – Intern 2 - “I personally have a lot of anxiety from being put on stage.” – Student 2b
Strategies for Adaptation	- “I think it is still a little bit disorganized in some ways. And part of it is because we have not really forgotten our old habits and we’re trying to do new things. If we just forgot the old habits and we transitioned to new stuff, it would be easier.” – Resident 2 - “We had to figure out how to involve the med student process of learning into our rounding, so we did table rounds in the morning before in order to still have our presentations and our feedbacks.” – Student 2b - “We had to budget time more for the patients that [were] not getting the Collaborative Care. [For] the first few days, it was like how do we cover these patients that we’re not seeing, and we had to figure out the time. And we actually have to come in earlier. Like we round with the attending at an earlier time than the other teams.” – Student 2b - “I think [Dr. A] was really good at doing that. […] [Dr. A] took the initiative to say ‘You know what, we’re going to step back a second, I am going to do some teaching.’ […] And she is really natural when she tells the patients that. It was really good and I mean I got a lot more from those rounds. I was able to learn on the spot in the room with the patient.” – Student 4
Improved Communication with Patients and the Team	- “Eventually like we are going to be the people who are leading the conversations, when we actually do become doctors. So I think it kind of puts you in a position where you have to lead the conversation sooner than in other rotations or in other internal medicine experiences that I have had. Because there are some times where I will say everything to the attending outside the room, but then when we go in, I don’t say anything. So it gives you the opportunity to speak, interact with the patient.” – Student 5 - “I have learned […] mostly about what is important to people and what is important for the family, what is important for the patient, and how to communicate with all the members of the team, like the nursing staff. And how, I guess that is something that surprised me a lot, how I can rely on the nursing staffs more […]. I think before I never felt supported by the nursing staff and now I do, so that surprised me.” – Resident 2 - “It makes you [a] more cognizant educator as far as teaching the patient about their condition and being able to kind of dematerialize the plan into something that they can cogently […] deal with. So that is the one way it impacts you know my learning. It is more about interpersonal learning and […] just being more aware how you see things.” – Student 3

*Loss of Educational Opportunities during Rounds* reflects learners’ perceptions of fewer teaching opportunities during collaborative, interprofessional bedside rounds when compared with the traditional inpatient physician rounds. Time spent in interprofessional discussions was perceived as less time for formal presentations and discussions of differential diagnoses and general medical topics. An outgrowth of this perception was also a feeling that they received less feedback in these areas (e.g., pointers on diagnostic and management strategies). Medical residents noted fewer opportunities for medical students to “shine” during formal presentations of traditional rounds. One student described how he preferred learning how to manage illnesses over learning how to work with the caseworker.

*Feelings of Uncertainty in New Situations* was a second theme*.* Learners expressed feeling uncertain in a variety of ways, with these instances generally occurring during interprofessional bedside rounds. One manifestation related to not knowing how to address sensitive topics (e.g., potential new diagnosis of cancer) in front of patients during bedside rounds. Learners were not only uncertain about what they should say in front of the patient, but also what topics (medical or otherwise) were appropriate to ask or explore further in an IPCP context. Another area of uncertainty, related to the loss of educational opportunities in the first theme, was whether or not to interrupt the flow of conversation to clarify a point or ask questions. Related, learners reported refraining from asking detailed questions about the plan of care during rounds because they did not want the patient to feel that there was not a unified team plan. Lastly, one learner described feeling anxious during interprofessional rounds because of the larger size of the team, likening her anxiety to “stage fright.”

*Strategies for Adaptation* reflected learners’ recognition that adapting to a different rounding style and overall workflow was difficult and that deviating from the familiar patterns was required. They pointed to team flexibility as an adaptive response, often enumerating examples of how the team adapted to respond to perceived needs. In one case, a team changed its workflow by reincorporating separate parallel “card flipping” rounds with more traditional physician-focused presentations prior to collaborative rounds. Other frequent examples of adaptation included the attending physician intentionally creating new learning opportunities within collaborative rounds or adding formal teaching sessions to other parts of the day. One learner described how an attending physician explicitly announced that “teaching” was occurring at the patient’s bedside before bringing up a medical teaching point.

Almost all medical learners commented on the theme of *Improved Communication with Patients and the Team*. Many learners acknowledged that CC provided more opportunities for communication with patients, patients’ family members, and other team members. They noted that collaborative rounding helped them to stay engaged with patients other than their own, as well as to practice communicating directly with patients. One student recognized that communication with patients is an important skill; she described how CC enabled her to practice her communication skills with her patients sooner than in other non-CC rotations. Learners also described how the CC model increased the quality and frequency of their communication with other professions and ultimately enhanced interprofessional trust. Some learners explicitly stated that the ability to practice communication skills with patients and team members is an educational opportunity unique to the CC IPCP model.

## Discussion

Incorporating medical learners into IPCP settings is complex. While our medical learners recognized and embraced improved communication between providers, patients, and families, they reported concerns related to teaching and learning in this model in three of four themes.

Medical learners reported feelings of uncertainty, particularly during collaborative rounds. They were unsure of what to ask or say, or what topics (e.g., a new cancer diagnosis) were appropriate or inappropriate for discussion at patients’ bedsides. Concerns and difficulties with rounding at patients’ bedsides are not a new phenomenon for learners [22], and bedside rounding is not always the norm at our institution. It is possible that some of this uncertainty was unrelated to the interprofessional component of our IPCP model and would have been present with bedside rounding on our other teams as well. In either case, techniques that have been reported to increase learner comfort at the bedside may be helpful in IPCP environments. These techniques include clarifying roles and expectations, emphasizing the importance of communication and interpersonal skills, and simply giving learners more experience [14, 23]. Targeted educational activities that make implicit uncertainty explicit, and offer ways for learners to work through this uncertainty, could be especially useful educational innovations in collaborative practice environments.

As mentioned, collaborative rounds are by definition bedside rounds, but they also incorporate interprofessional communication, dynamics, and different profession-specific priorities. Collaborative rounds are so novel that robust workplace-based learning strategies and relevant approaches to faculty development have not yet evolved, and this may account for some of the unfavorable effects on learning noted. For instance, medical learners perceived unfavorable effects on profession-specific education. Specifically, medical learners noted fewer opportunities to engage in typical clinical workplace-based learning activities such as formal verbal presentations (e.g., Subjective, Objective, Assessment and Plan or SOAP) during rounds and less discussion of principles underlying medical decision-making. Medical learners did not view their contributions (e.g., participation in bedside discussions with interprofessional team members, leading interprofessional discussions, and/or integrating and writing the team’s plan on the white board) during rounds as equally valuable.

The numerous adaptions our medical learners reported often involved residents and/or faculty attempting to recreate aspects of traditional teaching and learning within this new IPCP environment, such as re-incorporating more profession-specific verbal presentations and discussions into the day and dedicating specific time for profession-specific educational activities. From a workplace learning perspective, these reported adaptations represent the beginning stages of laying down new educational traditions in both interprofessional, and profession-specific teaching and learning. While it is natural to try to return to prior habits, educators should resist the urge to make every traditional educational experience fit into IPCP models, while actively identifying opportunities for novel pedagogies to introduce profession-specific content. This is also an area where formal educational curricula can both supplement and inform emerging workplace based interprofessional learning.

Although not a theme identified from our learner interviews, it is important to recognize that potential misalignment of assessments likely influence our findings. While we have chosen workplace learning as the ideal lens through which to examine our findings, more formal educational oversight and assessment strategies govern both undergraduate and graduate medical education. Our medical learners are familiar with UME [24] and GME [25] milestones and competencies. While these competencies include both profession-specific and interprofessional descriptions of knowledge, skills and attitudes, our medical learners’ responses indicate that they may not be as familiar with interprofessional competencies, or perhaps may not value them as much. As currently conceived, interprofessional competencies align with and complement profession-specific competencies primarily in areas of communication and teamwork [26]. Our medical learners may prioritize other profession-specific competencies (such as medical knowledge) more at this stage of their professional development. We speculate that this asymmetry in importance assigned to medical knowledge, at the expense of other domains of competence, is reinforced by the importance assigned by clerkships to National Board of Medical Examiners exams [27] and by residencies with specialty-specific licensing exams. In addition, current conceptualizations of interprofessional competency do not always make acquisition of tacit, or informal, knowledge explicit in the same way it does profession-specific knowledge. To address this discordance, assessment strategies for medical learners in IPCP environments will need to reflect attainment of both profession-specific and interprofessional competencies.

Our study has limitations. We report on medical learners in a single institution, and medical learner responses may differ at other institutions. For our initial analysis, we did not stratify by learner level – future studies should consider analyzing by learner experience to see if findings are consistent across training. Also, as noted, we included only medical learners in this analysis, as we did not have a sufficient number of learners from other professions to assess their experiences. Although we would expect learners across professions to struggle with the potential loss of profession-specific experiences, including learners from non-medical professions should be prioritized in future studies of inpatient IPCP clinical learning environments.

## Conclusions

Our results provide important insights from medical learners’ experiences on hospital-based IPCP teams. Medical learners reported loss of educational opportunities and felt uncertain in various contexts, while noting team adaptation strategies and improved communication. These insights are important to health professionals and educators who wish to engage medical learners in IPCP environments.

## Supplementary information

**Additional file 1:Supplementary file 1.** Interview questions. Contains the semi-structured interview questions that were used for participants.

## Data Availability

The datasets used and/or analyzed during the current study are available from the corresponding author on reasonable request.

## Abbreviations

CC: Collaborative care
IPCP: Interprofessional collaborative practice
IPE: Interprofessional education
UH: University Hospital
UTHSA: University of Texas Health San Antonio
