# Supplementary material for: Ready to collaborate?: medical learner experiences in interprofessional collaborative practice settings
Source: BMC Med Educ. 2020 Mar 23;20:85. doi: 10.1186/s12909-020-1992-1 (PMC7092524; doi:10.1186/s12909-020-1992-1)
Supplement: Supplementary file 1 — Additional file 1:Supplementary file 1. Interview questions. Contains the semi-structured interview questions that were used for participants. [file 12909_2020_1992_MOESM1_ESM.docx]

Supplementary file 1: Interview questions

Collaborative care

1. How was this rotation different from your other rotations? Probe with:
   1. Were your relationships with patients different?
   2. Were your relationships with other members of your team different?
   3. Were your relationships with other providers different?
2. What did you like/ not like about the experience?
3. What surprised you about the experience?
4. How did the model impact your learning?
5. How did the model change your activities throughout the day?
6. What would you/did you tell the person who will be replacing you about this experience and what they should expect?
7. Are there any specific tips or suggestions you would give to the(medical students OR Interns OR residents) rotating onto this team soon in regards to implementing a collaborative care model/ being on a collaborative care team?

        Possible ideas to prompt answers:

- 1. Adjusting to changes in workflow throughout the day?
  2. Using the communication board effectively during rounds (i.e. choosing what to write, summarizing)?
  3. Presenting directly to and interacting with patients and family during rounds (and outside of rounds)?
  4. Rounding efficiently and effectively?
  5. Interacting and communicating with other team members?
  6. Getting work done efficiently?
  7. Using the rolling computer during rounds for notes and orders?
  8. Communicating with family about patient’s care?
  9. Learning and teaching?
